# Ventilator-associated pneumonia in the era of COVID-19 pandemic: How common and what is the impact?

**DOI:** 10.1186/s13054-021-03571-z

**Published:** 2021-04-21

**Authors:** Paul-Henri Wicky, Michael S. Niedermann, Jean-François Timsit

**Affiliations:** 1 Medical and Infectious Diseases ICU (MI2), AP-HP, Bichat Hospital, 75018 Paris, France; 2 Department of Medicine, Weill Cornell Medicine, New York, USA; 3 Pulmonary and Critical Care Medicine, New York Presbyterian/Weill Cornell Medical Center, New York, USA; 4 IAME, INSERM, University of Paris, 75018 Paris, France

**Keywords:** COVID-19, ARDS, Ventilation-associated pneumonia, Superinfections, Prognostic

## Abstract

We reviewed similarities and differences of ventilator associated pneumonia in Sars-Cov2 infection and with other ARDS. The differences in epidemiology and outcome will be detailed. Possible explanations of differences in pathophysiology of VAP in Sarscov2 infections will be cited and discussed.

Addressing the common issue of antimicrobial stewardship for bacterial superinfections in severe SARS-CoV-2 infections is particularly challenging, especially with the uncertainties about how to diagnose ventilator-associated pneumonia (VAP) and tracheobronchitis (VAT). As compared to other viral pneumonias, the reported incidence of community-acquired pulmonary bacterial coinfections with COVID-19 is as low as 3% and 5–16% for ward and Intensive Care Units (ICU) patients respectively [1, 2]. However, the frequency of VAP is uncertain, and its incidence, characteristics and prognosis remain to be further explored (Fig. 1).

**Fig. 1:**
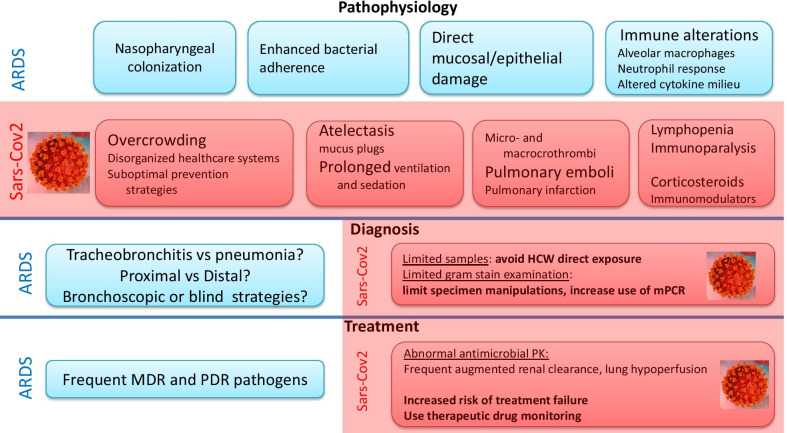
VAP risk in Sars-Cov2 infections and other ARDS: similarities and differences. The main elements of pathophysiology, diagnosis and treatment of VAP in ARDS patients are schematized in blue. In red, the potential elements due to sars-cov2 are discussed. *MDR* Multi-drug resistant; *PDR* pan-drug resistant; *PK* pharmacokinetic; *mPCR* multiplex PCR or other molecular methods

Significant disparities exist in the epidemiology of VAP, arguing for a standardization of definitions. Blonz et al., reported a crude incidence rate of 48.9% [1] in agreement with incidence rates ranging from 48 to 79% [3–6] in other cohorts. This rate contrasts substantially with the 29% observed in a multicenter study performed during the first COVID-19 wave in Italy [7]. In a retrospective study of 91 patients with COVID-19 respiratory failure (81 on a ventilator for > 48 h), Maes et al. reported a hazard ratio of 2.1, compared to non-COVID-19 patients, and an incidence of 79% with VAP [6]. In another study of 568 COVID-19 patients, 50.5% had either VAP or VAT, a higher incidence than was seen in influenza pneumonia or non-viral pneumonia [7]. It is clearly difficult to differentiate VAT from VAP in severe SARS-Cov-2 infections where modifications of chest radiographic infiltrates might be related to technical problems or intercurrent non-infectious events [8].

Additionally, VAP incidence may vary according to the bacteriological test used. Indeed, to avoid healthcare workers (HCW) contamination when the diagnosis of VAP is suspected, the use of bacteriological samples and bronchoscopy have been reduced, and gram stain examination not performed. The majority of VAPs were diagnosed based on bacteriological analysis from endotracheal aspirates (42.6%) in the study by Blonz et al., similar to the methods chosen by others [6]. Bronchoalveolar lavage (BAL) accounted for a quarter of sampling technique in COVID-ARDS patients, compared to 60% in non-COVID ARDS, in one study [3]. Importantly, quantitative distal as well as proximal samples were performed, but some authors only considered distal samples [3, 4], and others also included molecular methods [5, 9].

The increased risk of VAP in SARS-CoV-2 infections, as compared to other ARDS, may have been due to multiple factors including: less rigorous use of standard prevention strategies during COVID-19, disease and therapy-associated immune impairment, more prolonged duration of mechanical ventilation, prolonged use of sedation, more frequent need for prone ventilation, and higher risk for pulmonary infarction with associated superinfection. Although ICU overcrowding could also have been a factor, the study of Blonz et al. was done in an “uninundated” region where ICUs had adequate facilities for providing usual level of patient care, and thus there were less potential breaches in contact isolation. Similarly, in another single center study, a VAP rate reaching 74% was observed during both the first overcrowded wave and during the second wave where the ICU beds were sufficient [10].

SARS-CoV-2 ARDS patients have different clinical features than other ARDS patients, characterized by more profound hypoxia, and in comparative studies, the duration of mechanical ventilation was twice as long in COVID-19 patients compared with other types of ARDS [4, 5]. The extensive use of prone positioning could also have affected VAP incidence, and this therapy has been used in 67–83% of COVID-19 patients, generally twice as often as in influenza ARDS [3, 6]. Another difference with COVID-19 is the high risk of initial pulmonary emboli, which could predisposes to pulmonary infarction and secondary superinfections [11]. Immune alterations of the lung observed in ARDS patients [12] and COVID-19 patients, could be further amplified in SARS-CoV-2 infections by the use of corticosteroids or interleukin-receptor antagonists, as is suggested by some studies [13, 14], but not confirmed by recent randomized controlled trials [15].

VAP complicating SARS-CoV-2 infections occurred often late during mechanical ventilation [1, 4, 5]. Pathogens recovered are dependent on the local epidemiology. Enterobacterales accounted for two thirds of VAP (mainly *Escherichia coli* and *Klebsiella pneumoniae*), with half of these organisms being resistant to 3^rd^-generation cephalosporins. Notably, a significantly higher rate of Extended Spectrum Beta-lactamase-producing Enterobacterales (ESBL-PE) have been reported, compared to historical non-COVID-19 controls (72% vs 47%), and Aspergillus appears more common in COVID-19 respiratory failure than in other populations [3]. Interestingly, bloodstream infections (BSI) occurred in 10.6% of cases while pneumonia represented 21% of the source of BSI. Although prior antibiotic therapy can generally predispose to resistant organisms, Blonz et al. found that initial empiric therapy seemed to reduce the risk of polymicrobial VAP [1].

Finally some studies found an important rate of complicated VAP with lung abscesses and empyema [1] Substantial perfusion defects and impaired antibiotic diffusion into the parenchyma, could reasonably explain these failures, due to insufficient antibiotic concentration in the lung. We think that close therapeutic drug monitoring should be used in these patients with augmented renal clearance, to prevent therapeutic failures [12]. Given the high risk of pulmonary superinfections, and antibiotic failure, considerable effort to promote and implement prevention policies are of key importance especially in case of pandemic and healthcare system overcrowding. In this particular situation, even if MDR bacteria colonization is frequent, selective digestive or oral decontamination, or early intravenous antibiotic prophylaxis might be tested.

## Data Availability

Not applicable.

## Abbreviations

ICU: intensive care units
VAP: ventilation-associated pneumonia
ARDS: acute respiratory distress syndrome
VAT: ventilation-associated tracheobronchitis
HCW: health care workers
BAL: bronchoalveolar lavage
ESBL-PE: extended spectrum beta-lactamase producing enterobacterales
BSI: blood stream infections
MDR: multidrug resistant
PDR: pan-drug resistant
PK: pharmacokinetic
mPCR: multiplex PCR or other molecular methods
